# Oxalate induces type II epithelial to mesenchymal transition (EMT) in inner medullary collecting duct cells (IMCD) *in vitro* and stimulate the expression of osteogenic and fibrotic markers in kidney medulla *in vivo*

**DOI:** 10.18632/oncotarget.26634

**Published:** 2019-02-01

**Authors:** Marcia Convento, Edson Pessoa, Alef Aragão, Nestor Schor, Fernanda Borges

**Affiliations:** ^1^ Nephrology Division, Department of Medicine, Federal University of São Paulo, São Paulo, SP, Brazil; ^2^ Interdisciplinary Postgraduate Program in Health Sciences, Universidade Cruzeiro do Sul, São Paulo, SP, Brazil

**Keywords:** epithelial to mesenchymal transition, osteogenic differentiation, oxalate, TGF-β1, renal medulla

## Abstract

EMT occurs in response to a number of stresses conditions as mechanical stretch, cancer, hypoxia, oxidative stress (ROS), among others. EMT describes a phenotypical change induced in epithelial cells. It is characterized by increases in motility, extracellular matrix synthesis, proliferation, and invasiveness. The present study analyzed if oxalate ions (Ox) could induce EMT in IMCD cells. Ox (0.5 mM) and transforming growth factor beta (TGF-β1 20 ng/mL) exposition during 48 hours increased migration and invasiveness, increased mesenchymal marker expression (Vimentin, alpha-smooth muscle actin: α-SMA, TGF-β1) and decreased epithelial marker expression (E-cadherin). IMCD stimulated with Ox and TGF-β1 and then exposed to the osteogenic medium during 15 days significantly increased early osteogenic markers (RUNX-2 and Alkaline Phosphatase) expression. Hyperoxaluric mice fed with trans-4-hydroxy-L-proline (HPL) presented calcium oxalate crystal excretion, increased in TGF-β1 expression and collagen fibers deposition and increased early osteogenic markers (RUNX-2 and Alkaline Phosphatase) at 60 days. Our *in vitro* and *in vivo* results suggest that oxalate induces EMT in inner medulla collecting duct cells and it may be involved in fibrotic tissue development, osteogenic differentiation and calcium crystal production both implicated in nephrolithiasis.

## INTRODUCTION

Oxalate (Ox) is a regular by-product of metabolism and the small amounts produced are generally harmlessly excreted in the urine. Nevertheless, increased urinary excretion of oxalate (Ox) as the result of either genetic (primary hyperoxaluria) and environmental factors (Idiopathic hyperoxaluria), oxalate-rich foods ingestion (secondary hyperoxaluria), fat malabsorption due to jejunal bypass surgery and modern gastric bypass (enteric hyperoxaluria) [1, 2] can lead to urolithiasis, nephrocalcinosis, pyelonephritis, hydronephrosis, fibrosis and renal failure [3, 4].

Manifold animal models have been developed to investigate hyperoxaluria and its consequences. In the ethylene glycol [5] and HPL [3] induced hyperoxaluria animal model, it was found serious damage to the tubulointerstitial area. These lesions were characterized by tubular epithelial cell necrosis, calcium oxalate crystal deposits in tubular lumens, and inflammatory infiltrates, lipid peroxidation and proliferation of resident interstitial cells, such as fibroblasts and also, by an increase in extracellular matrix components including collagens fibers [1, 6–8].

Oxalate has been shown to be toxic in renal epithelial cells of cortical origin [9–13]. However, inner medullary collecting duct (IMCD) cells that are physiologically exposed to higher concentrations of oxalate can behave differently. These cells survive in a unique environment within the organism, commonly exposed otherwise lethal extremes of osmolality, pH, and toxins. Maroni *et al*. [14], demonstrated that porcine proximal tubular cells (LLC-PK1) and human renal proximal tubular cells (HK-2) were significant injuried at lower sodium oxalate concentrations compared with IMCD cells. Additionally, Brady *et al*. [15], suggested that HK-2 cells are more sensitive than IMCD cells to cisplatin cytotoxicity *in vitro*, confirming the idea that IMCD cells are relatively more resistant to toxicity. Nevertheless, IMCD cells may not be inert to oxalate effects and could participate in nephrolithiasis through of phenotype transitions and osteogenic acquiring characteristics, as recently shown in hyperoxaluric mice *in vivo* [16].

Another study of our group demonstrated that oxalate ions exposition to human proximal tubular epithelial cells (HK-2) stimulated the type 2 epithelial to mesenchymal transition (EMT) [17].

The type 1 EMT occurs during normal organogenesis. Type 2 EMT is associated with wound healing, tissue regeneration, and organ fibrosis. Type 3 EMT is related to neoplastic cells can migrate into surrounding tissues and invade at metastasis sites [18–20].

The type 2 EMT is an essential manifestation of epithelial cell plasticity during tissue regeneration, and organ fibrosis, it is associated with tissue repair responses such as fibrosis to underlying injuries in organs. In renal fibrosis if the injury is mild and acute, the healing process is regarded as reparative fibrosis; on the contrary, in ongoing chronic inflammation, abnormal formation of myofibroblasts, characterized by increased motility, extracellular matrix protein synthesis, proliferation, and invasiveness [18–20].

Losses of epithelial cell markers expression occur concomitantly with, and as a driver of, these changes. The architecture and permanent of epithelial cells, in sealing of tight junctions, depend on cell-cell contacts containing E-cadherin. Besides supporting cell-cell adhesion, cadherins can affect a wide range of cellular functions that include activation of cell signaling pathways, regulation of the cytoskeleton and control of cell polarity [18–20].

Transdifferentiated epithelial cells lose their defined cell-cell-basement membrane contacts and their structural/functional polarity to become spindle-shaped and morphologically similar to activated fibroblasts, there are mesenchymal markers expressed in type 2 EMT, as α-SMA which is a microfilament considered a marker of myofibroblast. Another mesenchymal marker shown is Vimentin, an intermediate filament whose expression is directly linked to cellular phenotypic changes [18–20].

Type 2 EMT is believed to occur in response to some stressful environmental stimulus as mechanical stretch [21], toxicity by cyclosporine treatment [22], exposure to advanced glycation end products (AGE-consequence of hyperglycemia) [23], and oxidative stress [24].

Some soluble growth factors, cytokines, and extracellular proteins have been shown to affect type 2 EMT and to influence renal disease progression. Transforming growth factor beta (TGF-β1), however, appears to play a prominent role, with an increase in expression almost universally in progressive forms of renal disease [25, 26]. In fact, TGF-β1 can induce a genetic program of cell plasticity that involves key pathways and regulators of epithelial dedifferentiation, cytoskeletal reorganization, and proliferation. TGF-β1 induces expression of fibrotic genes and mediates glomerular and tubular cell apoptosis, the mechanism by which tubular epithelial cells may acquire a myofibroblastic phenotype essential for the pathogenesis of fibrosis [27–29].

The type 2 EMT has already been demonstrated in IMCD cells *in vitro*, through exposure with epidermal growth factor receptor [30], Bestrophin-1 [31], TGF-β1 [32], insulin-like growth factors [32], and growth differentiation factor-11 [27]. *In vivo* experiments showed that the congenital urinary tract obstruction in humans [33] and animals [34] in collector duct epithelial cell injury and type 2 EMT. However, the oxalate induction of type 2 EMT in the medullary collecting duct, which is not originated from mesenchymal metanephric but from ureteric bud, was not demonstrated.

Hyperoxaluric rats increased mesenchymal markers and osteogenic marker genes both in kidney cortex and medulla [16]. Thus, this study suggest that oxalate ions can induce type 2 TEM in IMCD cells and that these transformed cells could be stimulated to express osteogenic markers *in vitro*. The effects of oxalate *in vivo* on the renal medulla of mice will also be evaluated.

## RESULTS

To determine whether IMCD cells exposed to Ox and TGF-β1 had become more active in invasion than control cells, we evaluated this characteristic of mesenchymal cell using the transwell chamber assay.

**Figure 1:**
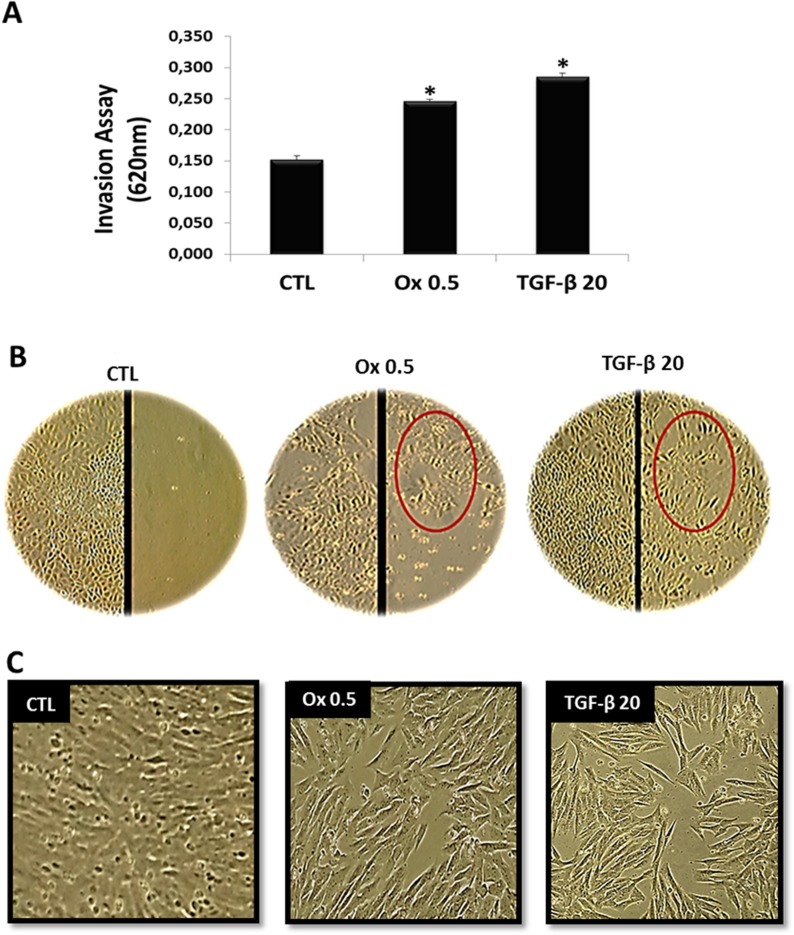
Characteristics of epithelial to mesenchymal transition *in vitro* (**A**) The invasion ability of IMCD cells exposed to culture medium (control), Ox (0.5 mM), and TGF-β1 (20 ng/mL) were examined as described in methods. (**B**) Representative light microscopic images show increased migration into a wound line of IMCD exposed Ox (0.5 mM) and TGF-β1 (20 ng/mL) in comparison to control cells. Epithelial cell culture showing monolayer growth with clear and rounded delimitations. Inversely, mesenchymal cells lose cellular adhesion and present long, tuned and scattered morphology (red circle). (**C**) Representative light microscopic images show morphological changes of IMCD cells; these cells stimulated with Ox and TGF-β1 are fusiform and much longer than control. Data are presented as means ± standard errors. (^*^) significant different when compared to the control group at *p* < 0.05. (ANOVA followed by a post hoc Tukey's test).

As shown in Figure 1A, IMCD cells exposed to Ox 0.5 mM (0.246 ± 0.003 DO) and TGF-β1 20 ng/mL (0.285 ± 0.006 DO) invaded through the pores faster than control cells (0.132 ± 0.005 DO). The acquisition of cellular invasion capacity is a characteristic of type 2 TEM. The Ox (0.5 mM) group acquire this ability, as well as the TGF-β1 stimulated group (positive control), which is considered the main mediator of type 2 TEM [35, 36]. Taking it into account, these groups remained in our study model.

Cellular migration ability is another characteristic of type 2 TEM [18, 19]. Epithelial cells *in vitro* grow till confluence to form a monolayer relatively static because the contact with neighboring cells inhibits migratory signals. The wound healing assay can be used experimentally to visualize the migratory ability of the cells through scratching a track through the monolayer. Cells at the edge of the incision become migratory and move. IMCD cells exposed to Ox (0.5 mM) and TGF-β1 (20 ng/mL) were seen to migrate through the incision in greater numbers than control cells (Figure 1B).

Figure 1C shows the phenotypic changes in IMCD cells exposed to Ox (0.5 mM) and TGF-β1 (20 ng/mL) in comparison to the control situation. IMCD cells exposed to Ox changed its morphological features notoriously. Control cells have a very clear and round boundary, individual cells abutting on each other in a uniform array. Additionally, there are regularly spaced cell to cell junctions and adhesions between neighboring cells. Very differently, IMCD cells exposed to Ox 0.5 (mM) and TGF-β1 (20 ng/mL) have a much longer and irregularly scattered cell shape, varying in density like mesenchymal cells.

**Figure 2:**
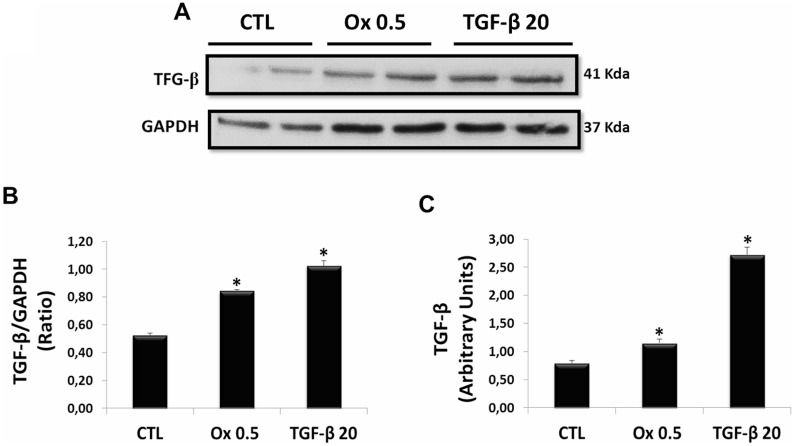
Evaluation of main mediator of epithelial to mesenchymal transition: endogenous synthesis of TGF-β1 *in vitro* (**A**) Western blot analyses in IMCD cells exposed to Ox (0.5 mM) and TGF-β1 (20 ng/mL) for expression of TGF-β1. (**B**) Quantitative analyses of immunoblot images were obtained by ImageJ software. (**C**) Real-time PCR showing the mRNA levels in IMCD using Syber Green assays for TGF-β1. Data are presented as means ± standard errors. (^*^) significant different when compared to the control group at *p* < 0.05. (ANOVA followed by a post hoc Tukey's test).

TGF-β1 acts as a potent driver of induction of Type 2 EMT [35, 36], and their endogenous synthesis is increased in chronic kidney diseases [25, 26]. There was a significant increase in expression of TGF-β1. The Figure 2A and 2B show the protein synthesis and quantitative analyses of immunoblot images (CTL: 0.53 ± 0.01 ratio, Ox 0.5 mM: 0.85 ± 0.01 ratio and TGF-β1 20 ng/mL: 1.02 ± 0.05 ratio), respectively. Figure 2C shows the gene expression (CTL: 0.80 ± 0.05 arbitrary units, Ox 0.5 mM: 1.15 ± 0.07 arbitrary units and TGF-β1 20 ng/mL: 2.72 ± 0.13 arbitrary units). Additionally, it is interesting to note that in our experimental conditions exogenous TGF-β1 was able to stimulate its endogenous gene expression and protein synthesis, amplifying its signal in a “positive feedback” loop.

**Figure 3:**
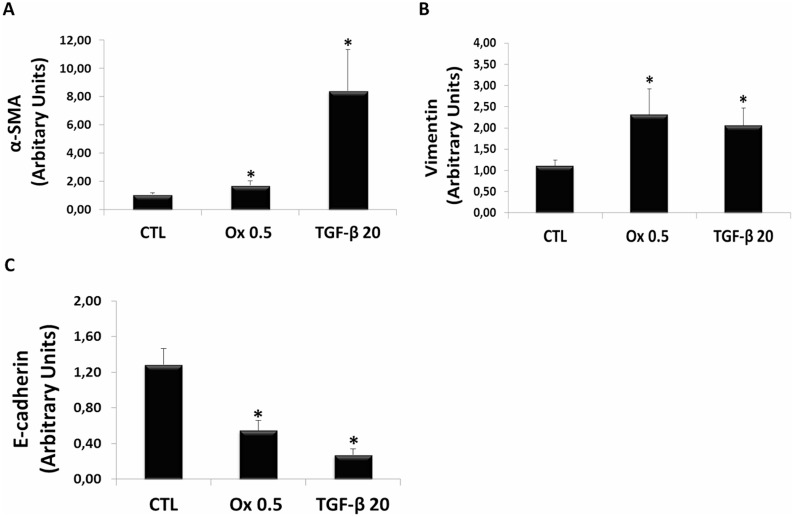
Markers of epithelial to mesenchymal transition *in vitro* Real-time PCR showing the mRNA levels in IMCD exposed to control situation, Ox (0.5 mM) and TGF-β1 (20 ng/mL) using Syber Green assays for α-SMA (**A**), Vimentin (**B**), and E-cadherin (**C**). Data are presented as means ± standard errors. (^*^) significant different when compared to the control group at *p* < 0.05. (ANOVA followed by a post hoc Tukey's test).

Figure 3A and 3B show the gene expression of mesenchymal cells markers. There was a significant increase in mesenchymal markers in IMCD cells stimulated with Ox (0.5 mM) including α-SMA (1.72 ± 0.31 arbitrary units), and Vimentin (2.31 ± 0.61 arbitrary units) compared to the control situation for α-SMA (1.06 ± 0.11 arbitrary units) and Vimentin (1.11 ± 0.13 arbitrary units). The stimulation with TGF-β1 (20 ng/mL) also induced a significant increase in α-SMA (8.40 ± 2.95 arbitrary units) and Vimentin (2.06 ± 0.41 arbitrary units) expression.

Figure 3C shows the gene expression of epithelial cell marker. There was a significant decrease in epithelial marker E-cadherin in IMCD cells stimulated with Ox (0.5 mM) (0.55 ± 0.11 arbitrary units), compared to the control situation for E-cadherin (1.28 ± 0.18 arbitrary units). The stimulatio with TGF-β1 (20 ng/mL) also induced a significant decrease in E-cadherin expression (0.27 ± 0.07 arbitrary units).

**Figure 4:**
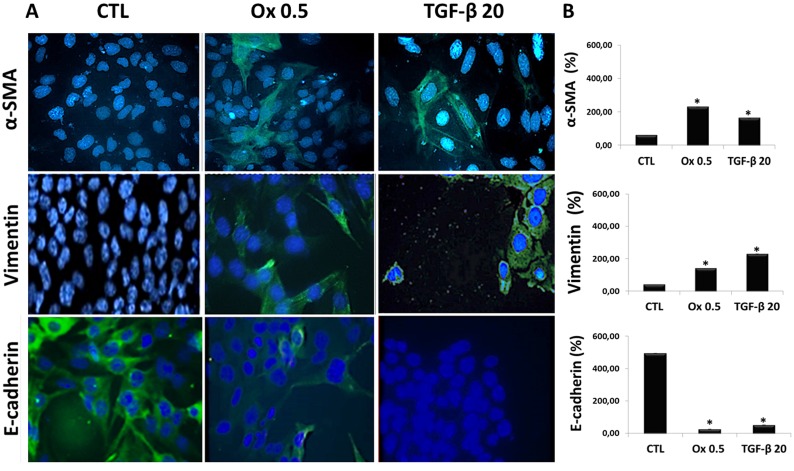
Markers of epithelial to mesenchymal transition *in vitro* (**A**) Immunofluorescence images (FITC: green fluorescence and blue: nuclei) showing α-SMA, Vimentin and E-cadherin immunostaining in IMCD cells exposed to Ox (0.5 mM) and TGF-β1 (20 ng/mL). (**B**) Quantitative analyses of immunoflorescence staining were obtained by ImageJ software. Data are presented as percentage. (^*^) significant different when compared to the control group at *p* < 0.05. (ANOVA followed by a post hoc Tukey's test).

Figure 4A shows the protein synthesis (immunofluorescence assay) for mesenchymal and epithelial markers. Ox (0.5 mM) and TGF-β1 (20 ng/mL) exposition for 48 h increased mesenchymal markers as α-SMA, Vimentin and decreased epithelial marker as E-cadherin in IMCD cells when compared to control situation. The Figure 4B shows the immunofluorescence quantification for α-SMA (CTL: 59.60 ± 0.41%, Ox 0.5 mM: 229.07 ± 0.35% and TGF-β1 20 ng/mL: 162,10 ± 0.35%), Vimentin (CTL: 39.18 ± 0.50%, Ox 0.5 mM: 139.78 ± 0.51% and TGF-β1 20 ng/mL: 228.82 ± 0.50%), E-cadherin (CTL: 493.52 ± 0.41%, Ox 0.5 mM: 25.26 ± 0.35% and TGF-β1 20 ng/mL: 50.28 ± 0.50%). The protein synthesis results corroborate our findings in gene expression. Nevertheless, the same effects were not observed after 48 h exposure to oxalate (1.0 mM) (Supplementary Figure 1), mainly because higher concentration of oxalate is toxic to IMCD cells.

**Figure 5:**
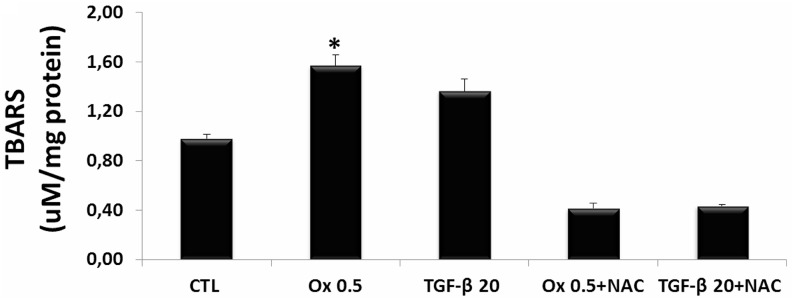
Evaluation of reactive oxygen species *in vitro* Quantitative analyses of thiobarbituric reactive substances (TBARS) in IMCD cells exposed to Ox (0.5 mM) and TGF-β1 (20 ng/mL) in the presence or absence of NAC (10 mM). Data are presented as means ± standard errors. (^*^) significant different when compared to the control group at *p* < 0.05. (ANOVA followed by a post hoc Tukey's test).

ROS is reported to be involved in cell injury and tight junction disruption [12]. The Figure 5 shows a significant increase in levels of lipid peroxidation (A) in IMCD cells stimulated with Ox (0.5 mM: 1.63 ± 0.05 μM/mg). Although TGF-β1 (20 ng/mL) seems to increase it, the difference was not significant (1.46 ± 0.12 μM/mg) in comparison to the CTL (1.38 ± 0.04 μM/mg) group. Conversely, cells that were stimulated with Ox or TGF-β1 and concomitantly treated with N-acetyl-L-cysteine (NAC) did not show increase in ROS.

**Figure 6:**
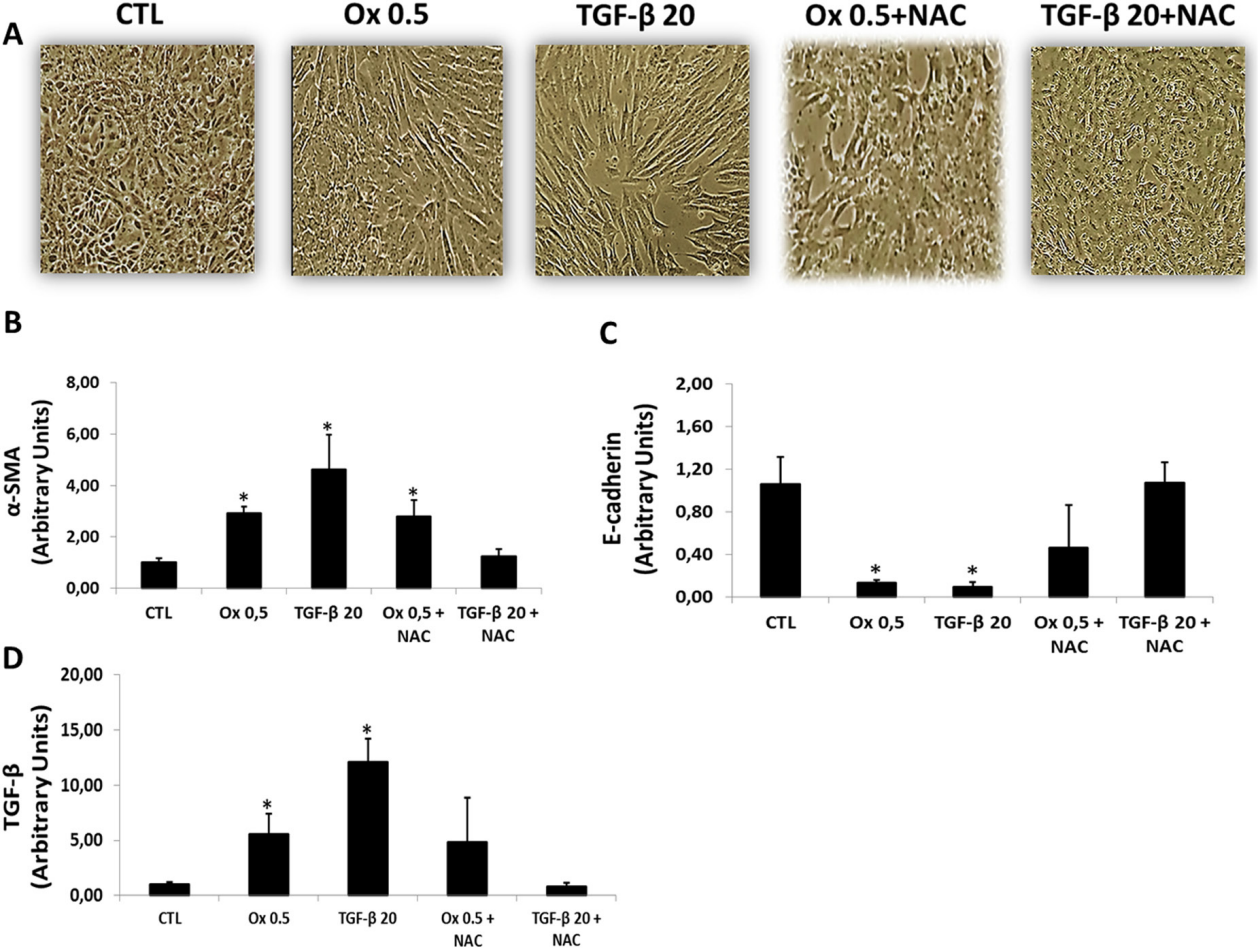
Effects of NAC on TGF-β1 and oxalate-induced EMT Representative light microscopic images show morphological changes of IMCD cells (**A**). Real-time PCR showing the mRNA in IMCD cells exposed to Ox (0.5 mM) and TGF-β1 (20 ng/mL) in the presence or the absence of NAC (10 mM) using Syber Green assays for α-SMA (**B**), E-cadherin (**C**), and TGF-β1 (**D**). Data are presented as means ± standard errors. (^*^) significant different when compared to the control group at *p* < 0.05. (ANOVA followed by a post hoc Tukey's test).

Effects of NAC on TGF-β1- and oxalate-induced EMT were assessed by changes in cell morphology and expression of mesenchymal and epithelial markers. Control cells maintained in culture conditions without stimulation exhibited a rounded cobblestone appearance. After stimulation with Ox and TGF-β1, IMCD cells displayed increase in overall size, loss of cell-cell contacts, and assumed a fibroblast-like morphology. Cells treated with NAC in the presence of Ox and TGF-β1 retained their rounded shape and cobblestone appearance consistent with retention of the epithelial phenotype (Figure 6A). Interesting, TGF-β1 seems to stimulate IMCD transition more effectively in comparison to Ox.

The NAC effect on EMT markers in IMCD cells stimulated with Ox and TGF-β1 is presented in Figure 6B.

IMCD cells treated concomitantly with TGF-β1 and NAC did not present decrease in epithelial marker E-cadherin (1.07 ± 0.19 arbitrary units) and did not increase the mesenchymal marker α-SMA (1.13 ± 0.17 arbitrary units) in comparison to control group (E-cadherin: 1.06 ± 0.25 arbitrary units; α-SMA: 1.02 ± 0.15 arbitrary units). Interestingly, in response to NAC treatment, there was no significant difference in epithelial marker E-cadherin after Ox exposition (0.47 ± 0.40 arbitrary units) when compared to the CTL group (1.06 ± 0.25 arbitrary units). However, the mesenchymal marker α-SMA increased significantly (2.79 ± 0.54 arbitrary units) after Ox exposition when compared to the CTL group (1.02 ± 0.15 arbitrary units). Our results suggest that NAC was more effective in inhibiting TEM induced by TGF-β1 than by Ox.

In response to NAC treatment, the stimulation with Ox increased endogenous TGF-β1 (4.86 ± 3.99 arbitrary units), although it was not significantly different when compared to the CTL group (1.01 ± 0.22 arbitrary units). Nevertheless, the endogenous synthesis of TGF-β1 was dramatically reduced in cells exposed concomitantly with NAC and stimulated with the exogenous transforming growth factor (0.82 ± 0.30 arbitrary units). This result corroborates the epithelial and mesenchymal markers expression (Figure 6C).

Interestingly, NAC seems to blunt TGF-β1-induced EMT while only partially change oxalate-induced EMT.

To proper analyze the participation of oxalate induced EMT in osteogenic genes expression, we submitted IMCD cells to oxalate and TGF-β1 followed by exposition to osteogenic differentiation medium during 15 days.

**Figure 7:**
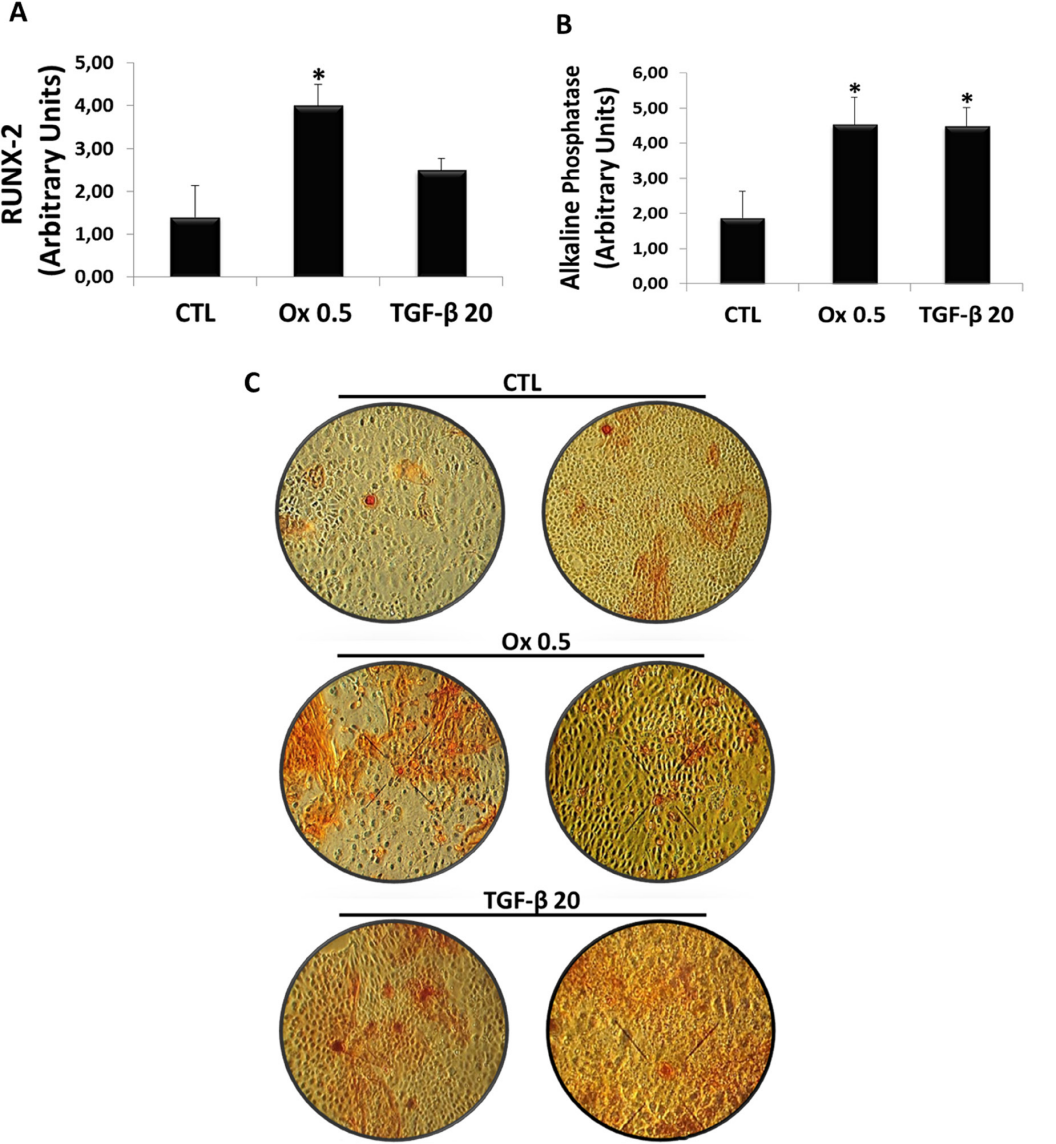
Evaluation of osteogenic genes expression *in vitro* Real-time PCR showing the mRNA levels in IMCD exposed to control situation, Ox (0.5 mM) and TGF-β1 (20 ng/mL) using Syber Green assays for RUNX-2 (**A**) and Alkaline Phosphatase (**B**). Image shows culture plates stained for extracellular matrix dye (red) in all groups (**C**). Data are presented as means ± standard errors. (^*^) significant different when compared to the control group at *p* < 0.05. (ANOVA followed by a post hoc Tukey's test).

The expression of osteogenic markers RUNX-2 (Figure 7A) and Alkaline Phosphatase (Figure 7B) were analyzed. For RUNX-2, Ox (0.5 mM) (3.99 ± 0.50 arbitrary units), significantly increased RUNX-2 expression in IMCD cells, while TGF-β1 (20 ng/mL) also increased but it was not significantly different (2.49 ± 0.18 arbitrary units) when compared to control (1.40 ± 0.74 arbitrary units). For Alkaline Phosphatase, both TGF-β1 (20 ng/mL) (4.48 ± 0.54 arbitrary units) and Ox (0.5 mM) (4.52 ± 0.67 arbitrary units) increased the osteogenic marker when compared to control situation (1.88 ± 0.65 arbitrary units). Figure 7C shows the osteocyte labeling (red) mainly in Ox and TGF-β1 groups.

**Figure 8:**
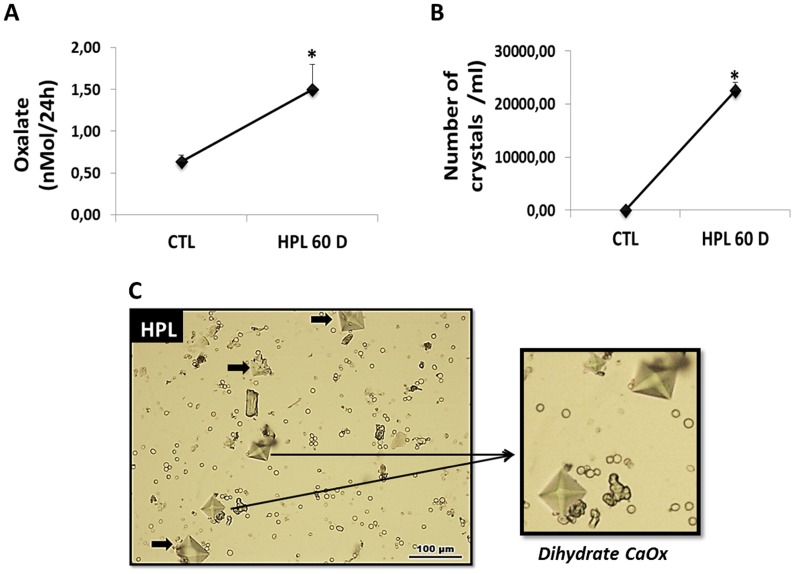
Evaluation of hyperoxaluria *in vivo* Animals fed with hydroxyproline (HPL) during 60 days (60 D) increased urinary oxalate excretion (**A**) and showed increase in urinary crystals number (**B**). Light microscopy images showing calcium oxalate crystals, with characteristics of CaOx dihydrate, observed in the urine of hyperoxaluric animals (**C**). Data are presented as means ± standard errors. (^*^) significant different when compared to the control group at *p* < 0.05. (ANOVA followed by a post hoc Tukey's test).

Hyperoxaluria can be induced by the administration of an agent such as HLP [3]. The results of our *in vivo* hyperoxaluric models are shown in Figure 8. Animals fed with HPL after 60 days had an increased excretion of Ox (Figure 8A) and presented calcium oxalate (CaOx) crystals in their urine (Figure 8B). Light microscopy images showed calcium oxalate crystals, mostly CaOx dihydrate crystals, observed in the urine of hyperoxaluric animals (Figure 8C).

**Figure 9:**
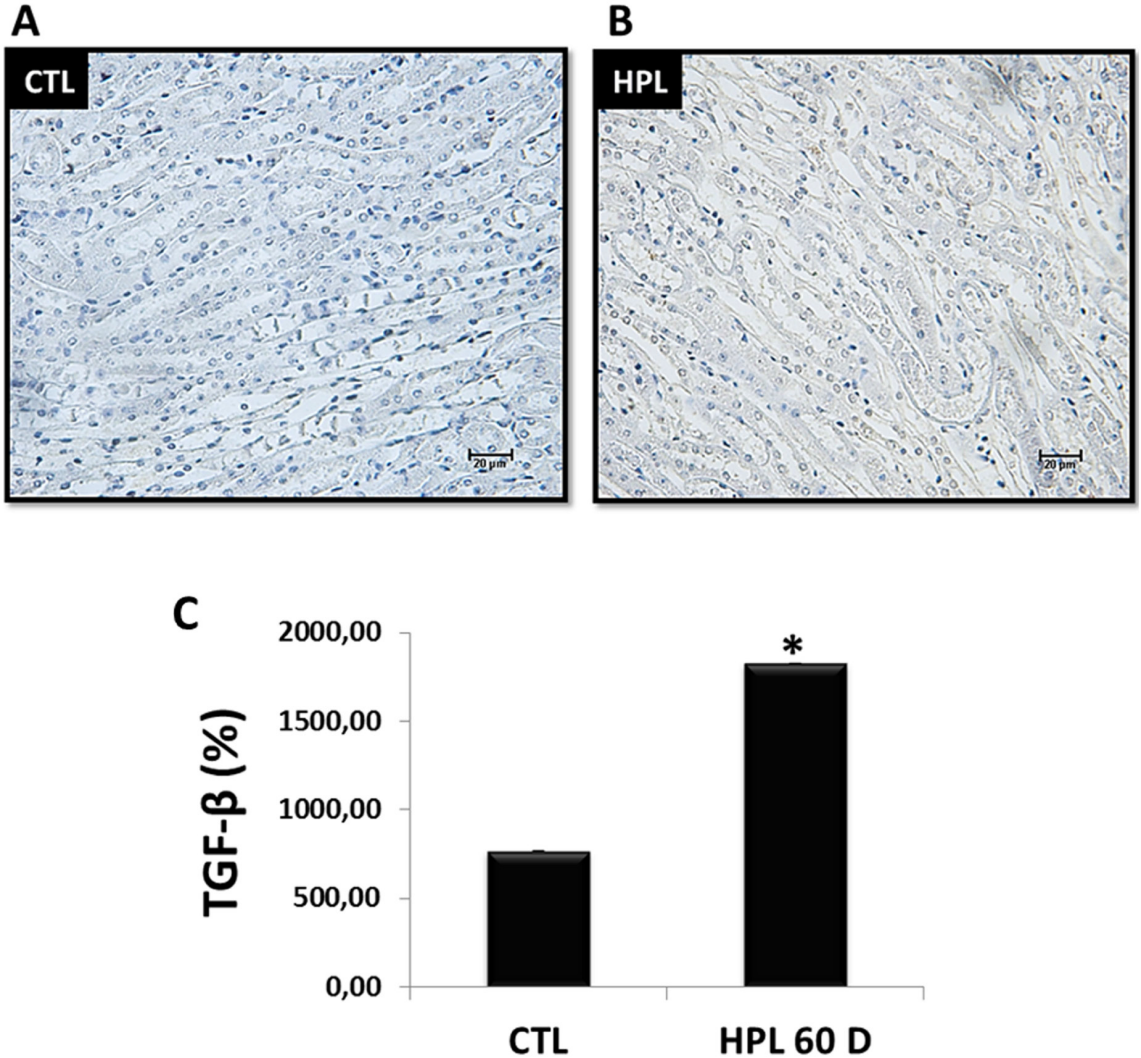
Evaluation of endogenous synthesis of TGF-β1 *in vivo* (**A–B**) The stimulation with hydroxyproline during 60 days (HPL 60 D) increased the expression of TGF-β1 according to immunohistochemistry image (grey tone). (**C**) Quantitative analyses of TGF-β1 were obtained by ImageJ software. Data are presented as means ± standard errors. (^*^) significant different when compared to the control group at *p* < 0.05. (ANOVA followed by a post hoc Tukey's test).

Figure 9A and 9B show immunostaining for endogenous synthesis of TGF-β1 *in vivo* at 60 days in renal medulla. We observed an increase in the labeling for TGF-β1 in the HPL group compared to the CTL group. The Figure 9C shows the quantification of immunostaining for TGF-β1 (CTL: 769.45 ± 0.65%, HPL: 1829.00 ± 0.50%), the main mediator of kidney fibrosis.

**Figure 10:**
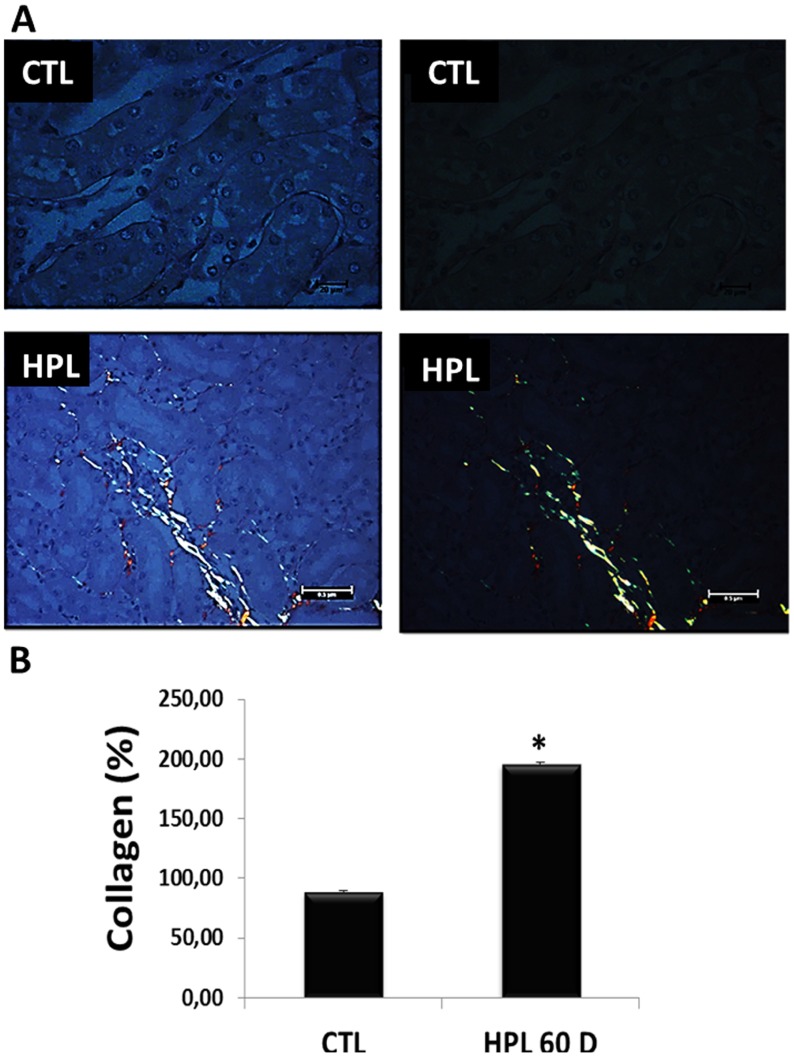
Evaluation of fibrotic marker *in vivo* (**A**) Animals fed with hydroxyproline during 60 days (HPL 60 D) increased the production of collagen type I (yellow to red tone) and collagen type III (greenish tone), showed by picrosirius staining. (**B**) Quantitative analyses of picrosirius staining were obtained by ImageJ software. Data are presented as means ± standard errors. (^*^) significant different when compared to the control group at *p* < 0.05. (ANOVA followed by a post hoc Tukey's test).

Corroborating the above result, hyperoxaluric mice treated with HPL increased the deposition of collagen fibers at 60 days (type I yellow to red tone and type III greenish tone) (Figure 10A) in the kidney medulla. The Figure 10B shows the graphical quantification for collagen (CTL: 88.68 ± 1.00%, HPL: 195.72 ± 1.49%).

**Figure 11:**
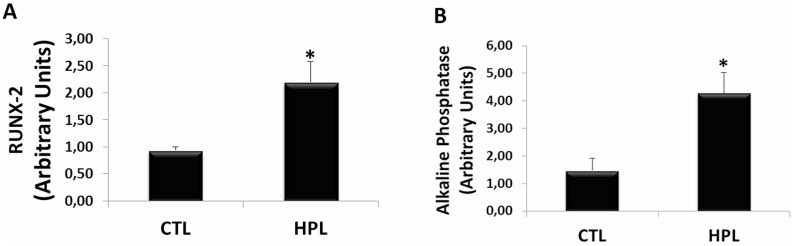
Evaluation of osteogenic gene expression *in vivo* Real-time PCR showing the mRNA levels of RUNX-2 (**A**) and Alkaline Phosphatase (**B**) in kidney medulla of animals fed with hydroxyproline (HPL) during 60 days (60 D). Data are presented as means ± standard errors. (^*^) significant different when compared to the control group at *p* < 0.05. (ANOVA followed by a post hoc Tukey's test).

Finally, we evaluated the participation of oxalate induced EMT in osteogenic genes expression in renal medulla. Hyperoxaluric mice treated with HPL for 60 days increased the expression of osteogenic genes *in vivo*. The expression of osteogenic markers RUNX-2 (Figure 11A) and Alkaline Phosphatase (Figure 11B) were analyzed. HPL (2.19 ± 0.18 arbitrary units) significantly increased RUNX-2 expression in comparison to control (0.94 ± 0.06 arbitrary units). For Alkaline Phosphatase, HPL (4.28 ± 0.74 arbitrary units) increased the osteogenic marker when compared to the control situation (1.47 ± 0.44 arbitrary units).

## DISCUSSION

In the present study, we demonstrate that inner medullary collecting duct (IMCD) cells stimulated with oxalate in comparison with TGF-β1, used as *in vitro* positive control, showed morphological changes, gaining migratory mesenchymal qualities, accompanied by a shift in the expression of epithelial genes to a mesenchymal gene repertoire, which is characteristic of cells undergoing Type 2 EMT [37, 38]. The type 2 EMT has already been demonstrated in IMCD cells *in vitro*, through exposure with TGF-β1 [29]. However, according to our knowledge, the oxalate induction was not demonstrated in literature.

The use of Ox stimulated endogenous synthesis of TGF-β1. Additionally, the exogenous stimulation with TGF-β1 in IMCD cells induced its own synthesis in a manner similar to a positive feedback loop, as reported by others [39–42, 62], confirming that TGF-β1 acts as a potent inductor of Type 2 EMT [35, 36]. Its synthesis is increased many experimental models of chronic kidney disease [25, 26], including our experimental conditions.

Based on changes in cell morphology, expression of a panel of mesenchymal and epithelial markers, and also through the endogenous synthesis of the major mediator of TEM, the TGF-β1, our findings suggest that NAC blunted TGF-β1-induced EMT. ROS production can act as second messengers to mediate the Type 2 EMT, but also inducing TGF-β1. This growth factor increases reactive oxygen species production and suppresses antioxidant enzymes, leading to a redox imbalance. Reactive oxygen species, in turn, induce TGF-β1 to mediate many its fibrogenic effects, forming a perverse vicious cycle for fibrosis [43].

Oxalate exposition is accompanied by the generation of hydrogen peroxide in renal cells [11, 12, 44], in agreement with our findings. In addition, hydrogen peroxide produced by mitochondria is the major member of ROS that is involved in tight junction disruption [45–47]. Other authors showed that the concomitant exposure to exogenous antioxidants N-acetylcysteine (NAC) attenuate the adverse effects of oxalate [12, 44] and TGF-β1 [48].

Nevertheless, in our conditions NAC only partially inhibited oxalate-induced EMT, suggesting that other mediators than ROS are involved in oxalate-induced EMT.

It is important to point out that our study has some limitations in understanding the effect of NAC in oxalate-induced EMT. Higher concentrations of NAC should be used since oxalate-induced EMT was more resistant to NAC effect than induced by TGF-β1. Other stimuli than ROS can be inducing TGF-β1, so further studies are necessary to clarify NAC effect in oxalate-induced EMT.

Other studies showed the relationship between TGF-β1 and transformation to osteogenic phenotype. Additionally, RUNX-2 and ALP are also common targets of TGF-β1 [49–51]. Since we observed that oxalate induced TEM and increased TGF-β1 in IMCD, to further test if type 2 EMT derived cells could differentiate into functional cell capable of producing mineral deposits, they were grown in osteogenic differentiation media for 15 days. Our data indicate that IMCD cells were able to express osteogenic gene like ALP, RUNX-2 and there was osteocyte staining as proven by Alizarin Red, which stain calcium deposits.

Different steps of osteogenic differentiation process including proliferation phase, extracellular matrix synthesis, and mineralization are detected by the expression of specific genes. The primary function of differentiated osteoblasts is the synthesis of type I collagen, the major constituent of extracellular matrix [16, 52], also involved in calcification [16, 53].

So, collagen is an early specific gene that increases in proliferative and in matrix maturation phases of osteogenesis. RUNX-2, called “a master gene,” is an essential transcription factor for the initiation of osteogenesis [54]. Several osteoblastic gene expressions, including type I collagen, are regulated by RUNX-2 at the early stages of osteoblast differentiation.

Alkaline phosphatase (ALP) is considered one of the most commonly accessible markers of osteoblast differentiation responsible for the mineralization of the extracellular matrix. The final stage results in production and release of aggregates containing calcium phosphate (CaP), also called hydroxylapatite [55–57].

The mixed CaP or hydroxylapatite and calcium oxalate (CaOx) represent approximately 80% of kidney stones [58]. However its pathogenesis is not fully understood. The most recent mechanism suggests that in some patients, stone formation probably involves the formation of Randall's plaque. Supersaturation of tubular fluid in collecting duct stimulates calcium oxalate crystallization. The renal papilla mineralizes through a mechanism not well understood, and a subepithelial plaque is established. The papillary surface epithelium is disrupted, the plaque rupture, attracting the deposition of CaOx crystals on the CaP base [59, 60].

In view of this, it is reasonable to suggest that oxalate induced Type 2 EMT and osteogenic transformation *in vitro* could be involved in nephrolithiasis.

Typically, in our conditions *in vitro*, osteogenic factors are introduced directly into the culture medium to drive the cells toward osteogenic differentiation. However, it is unlikely that these same factors (ascorbic acid, dexamethasone, and β-glycerol phosphate) are present within the natural environment *in vivo*, hence the need to expand the protocol for *in vivo* conditions.

Joshi et al. [16] showed that administration in rats with HPL for 28 days promoted up regulation of gene encoding for RUNX-2 during hyperoxaluric conditions and further increased after crystal deposition or nephrolithiasis, with down regulated of gene encoding for ALP. In view of this, we decided to double the period of hyperoxaluric stimulation with HPL in mice for 60 days.

In these conditions, we observed in renal medulla of hyperoxaluric mice the increase in type III collagen fiber deposition. It is associated with a higher degree of fibrosis [49], and is involved in pathogenesis of nephrolithiasis. The urine of hyperoxaluric mice also showed pyramid-shaped calcium oxalate dehydrate crystals (CaOx) and increase in Ox excretion, according to other publications [3, 61].

Additionally, we observed that the renal medulla acquired an osteogenic phenotype with upregulation of RUNX-2, ALP and collagen type I deposition. Our results suggest that endogenous synthesis of TGF-β1 *in vivo* induced by hyperoxaluria can also be involved in oxalate effect, as observed *in vitro*.

In conclusion, the study of phenotypic transitions improve our understanding of cell transformation involved in pathological processes such as renal fibrosis and nephrolithiasis. According to our knowledge, it is the first study showing that oxalate induce type 2 epithelial to mesenchymal transition in inner medullary collecting duct cells. The dedifferentiated cell under appropriated condition expressed osteogenic genes and positive osteocyte staining. Renal medulla of hyperoxaluric mice demonstrated upregulation of osteogenic genes and proteins involved in calcification as type I collagen, these upregulations suggesting the formation of calcium phosphate crystals that can serve as nidus to CaOx crystals growth. Oxalate *in vitro* and *in vivo* stimulates endogenous synthesis of TGF-β1 which is also involved in type 2 epithelial to mesenchymal transition and osteogenic differentiation, reflecting its pathogenic role in renal medulla, in our experimental conditions. Therefore, new studies are needed to clarify the numerous mechanisms involved in oxalate induced-EMT and its implication in nephrolothiasis.

## MATERIALS AND METHODS

### Cell culture

IMCD derived from mouse and presenting inner medullar collect duct phenotype was obtained from the American Type Culture Collection and grown in Dulbecco's modified Eagle's medium (DMEM, Sigma Chemicals, St. Louis, MO, USA) supplemented with 5% fetal bovine serum (FBS, Gibco, Carlsbad, CA, USA), 24 mM of NaHCO3, 10 mM of N’-2-hydroxyethylpiperazine-N’-2-ethanesulfonic acid, and 10,000 U/L of penicillin/streptomycin. Cells were grown to semiconfluence at 37°C in a humidified atmosphere containing 5% carbon dioxide. After this period, 0.5% trypsin (Cultilab, Campinas, Brazil) was then used to detach them from the flasks. The cells were subsequently centrifuged, resuspended in DMEM, and subcultured in 25-cm^2^ plastic culture flasks or 6 well plates for the experimental procedures.

### Preparation of oxalate ions

Solutions of dipotassium oxalate (0.4 M, 100 mL) (Merck, Darmstadt, Germany) were added to 300 mL of distilled, deionized water at a constant drip rate of 1 mL/min for 2 h. This suspension was stirred continuously for 5 h at 75°C and then washed with deionized water to remove the potassium chloride. Oxalate (10 mM) was added to phosphate-buffered saline (PBS- free calcium), sterilized in 0.22 μM filter and used in the respective protocols at the concentration of 0.5 mM (48 h). In the Supplementary Figure 1, the cells were exposed to 1.0 Mm (48 h), however, was not used in our study.

### Exposure of IMCD immortalized cells to oxalate ions (0.5 mM), exogenous TGF-β1 (20 ng/mL), and NAC (10 mM)

Before the experiments, immortalized cells (American type culture collection (ATCC)), were maintained under the culture conditions for 2 days. At confluence, IMCD were exposed for 48 hours to either DMEM (5% FBS, control), DMEM containing oxalate (0.5 mM), TGF-β1 (20 ng/mL, Peprotech, NJ, US), used as positive control of type 2 TEM, and N-acetylcysteine (10 Mm, Sigma, St. Louis, MO, USA). The experimental protocol was repeated at least 3 times at different periods. *N* = 5 for each group.

### Invasion assay

IMCD invasion was analyzed by trans-well assay; 10^5^ cells were added to the top chambers of 6-well trans-well plates (8 μm size, Millipore Corporation, Bilerica, MA, USA) and media containing 5% FBS with Ox and TGF-β1 was added to the bottom chambers for 48 h. Top (non-invasive) cells were removed, bottom (invasive) cells were fixed in formaldehyde 3.7%, stained with Trypan Blue, solubilized in sodium dodecyl sulfate (2%) and absorbance was recorded at 620 nm. All of these assays were done in triplicate.

### Immunofluorescence

IMCD cells were grown on Labtek II glass slides with 8 wells, then washed with PBS, fixed (3.7% fresh paraformaldehyde in PBS for 20 minutes at room temperature), permeabilized (0.5% Triton X-100 for 5 minutes), blocked with 5% albumin/phosphate buffered saline (BSA/PBS) for 60 min. Following blocking, primary rat monoclonal antibodies (Santa Cruz). 1:50 to E-cadherin, α-SMA and Vimentin were added overnight at 4°C in 0.5% BSA/PBS. The FITC-labeled secondary anti-rat IgG and anti-mouse IgG (1:100) (Santa Cruz) was added for 2 h at room temperature in 0.5% BSA/PBS and cells were incubated with DAPI (4′6-diamidino-2-phenylindole, Sigma) 10 μg/mL. Coverslips were mounted on glass slides with buffered glycerin and analyzed using Nikon fluorescence microscopy. The microscope images obtained were quantified using ImageJ software. The data are reported as percentage of positively fluorescence intensity per area [62].

### Migration assay

Wound healing assay is commonly used for assessing the effect of pro and anti-migratory agents on cultured cells [63]. Briefly, cells were grown to confluence in culture plastic dishes to a density of approximately 5×10^6^ cells/well. After 24 h of quiescence, the cells were denuded by dragging a rubber policeman through the center of the plate. Cultures were rinsed with PBS and replaced with fresh medium containing 5% FBS (control situation) and medium containing 5% FBS has added Ox (0.5 mM) and TGF-β1 (20 ng/mL), following which the IMCD cells were incubated at 37°C for 48 h and photographed.

### Real-time polymerase chain reaction (Real-time PCR)

Total Ribonucleic acid (RNA) was purified (IMCD cells and medulla renal of mice) by phenol and guanidine isothiocyanate-cesium chloride method using an appropriate kit (Trizol, Life Technologies, USA). 2 micrograms of total RNA were treated with DNase (RQ1 RNase-Free DNase, Promega) to prevent genomic deoxyribonucleic acid contamination. The RNA pellet was resuspended in RNase-free water. Reverse transcribed into cDNA by the addition of a mix containing 0.5 mg/mL oligo d (T), 10 mM DTT, 0.5 mM dNTPs (Pharmacia Biotech), and 200 U of reverse transcriptase enzyme (SuperScript RT, Gibco-BRL). Real-time amplification was obtained using a GeneAmp 7700 Sequence Detection System (SDS, ABI Prism 7700, Applied Biosystems) and monitored using the SYBR Green I intercalating dye (Applied Biosystems). PCR was performed with selective primers (Supplementary Table 1). Results were reported as a relative expression normalized with the β-actin housekeeping gene. The fold variation was determined using the 2-(ΔΔCt) method according to previously published protocol [64].

### Osteoinduction assay

IMCD cells were exposed for 48 hours to either DMEM (5% FBS, control), DMEM containing oxalate (0.5 mM) and TGF-β1 (20 ng/mL). After this period, cells were exposed in an osteogenic conditioned medium including α-MEM with 5% FBS, 50 μg/mL ascorbic acid (Gibco), 10 mM b-glycerol-phosphate (Gibco), and 10 nM dexamethasone (Sigma), for 15 days culture with daily change of osteogenic conditioned medium. Total RNA (Real-Time PCR) was extracted to detect the expression of osteogenic markers Runt-related transcription factor 2 (RUNX-2) and Alkaline Phosphatase (ALP). On day 15 was added the extracellular matrix dye (Alizarin Red S) on the plates in culture, and the images were photographed in all groups.

### *In vitro* peroxidation assay (Thiobarbituric acid reactive substances – TBARS)

The malondialdehyde (MDA) combines with thiobarbituric acid (TBA) forming a red compound, whose concentration was assessed by spectrophotometry with reading at 535 nm [65]. Culture medium without phenol and cell homogenate samples of cell cultures were stimulated with oxalate (0.5 mM) and TGF-β1 (20 ng/mL) in the absence and presence the NAC (10 mM) for 48 h. The supplementation with NAC provides sulphthiol group of L-cysteine and also exerts antioxidant effect by directly scavenging free radicals. Cellular homogenates were obtained by scrapping cells from the flasks with PBS. Samples were added to a solution of 0.375% TBA, 15% trichloroacetic acid, and 0.25 HCl (Sigma Chemicals), kept in continual agitation, heated in 95°C for 20 min and subsequently cooled to room temperature. The protein concentration was verified by the method of Lowry [66]. Positive control was obtained by incubating the cell samples with H_2_O_2_ at 1% for 1 h before the assay. The results were corrected through the protein and were expressed as μM/mg.

### Western blotting

The protein concentration was verified by the method of Lowry [66]. IMCD cells were lysed with a 200-μL RIPA lysis buffer per plate (100 mm^2^). The lysates were centrifuged at 12,000 g for 5 min at 4°C, and the supernatants were stored at −80°C. Proteins (30 μg) were separated by 10% polyacrylamide gel electrophoresis and transferred to polyvinylidene fluoride membranes using a Mini Trans-Blot Electrophoretic Transfer Cell (BioRad). Nonspecific binding sites were blocked with 5% BSA (v/v) in a TBS buffer. The immunoblots were incubated overnight at 4°C with the TGF-β1 and GAPDH primary antibodies (1:1000, Santa Cruz Biotechnology, Dallas, TX, USA). After washing three times with TBS-T, the membranes were incubated for 1 h at 4°C in HRP-conjugated secondary antibodies (1:30000; Cell Signalling). Immunoreactive protein bands were visualized using Pierce ECL Plus Chemiluminescent substrate detecting reagents (Thermo Fisher, USA). Images were obtained and analyzed with an Alliance 7 Chemiluminescence documentation system (UVItec, Cambridge, UK). The immunoblot band intensities were quantified using ImageJ software and expressed as the TGF-β1/GAPDH ratio.

### *In vivo* experimental groups

The experimental protocol was approved by the Ethics Committee of the Federal University of São Paulo (protocol number CEP 1098/10), also in agreement with the Brazilian guidelines for scientific animal care and use [67, 68]. The C57Bl/6 mice were divided into the following groups: a control group receiving water *ad libitum* for 60 days; an HPL group that received 5% HPL for 60 days *ad libitum*. At the end of the experimental protocol in 60 days, the mices were kept for 24 hours in metabolic cages for urine collection and were then sacrificed using a toxic dose of anesthetic (ketamine and xylazine). The kidneys were removed for histological analysis. The urine volume was measured to analyze of oxalate excretion. The experimental protocol was repeated 3 times at different moments with *N* = 5 for each group.

### Urine oxalate

Was analyzed with an oxalate oxidase kit (Trinity Biotech, Co. Wicklow, Ireland) following the manufacturer's protocol. The results were corrected through the urine volume and were expressed as mMol/24 h.

### Urine crystals

Urine sediment was obtained by centrifugation (2,300 rpm/min for 5 minutes) and counted in a Neubauer chamber following the formula: (final volume × number de crystals)/(initial volume × 0.0001) and was photographed. The results were expressed as urinary crystals/mL.

### Histochemistry assay

Paraffin sections were subjected to xylene and alcohol gradient solutions, antigen retrieval recovery, protein block, and incubation with primary rabbit anti-TGF-β1 (1:100, Santa Cruz, USA) overnight at 4°C. After this period, the sections were incubated with a dextran polymer conjugated to peroxidase antibody for 30 minutes (DAKO Envision kit, Dako, Denmark). Marking was detected by exposing the sections using a chromogenic substrate. The primary antibody was omitted as a negative control, and the sections were counterstained with hematoxylin and analyzed under a light microscope the Olympus BX 60 model at 20x or 40x magnification. The paraffin section of the kidney was also stained with picrosirius red and quantified under a polarized light microscope (Olympus BX 60) to evaluate the production of collagen types (type I yellow to red tone and type III greenish tone). The obtained microscope images were quantified using ImageJ software and expressed as a % of the stained area.

### Statistical analysis

The results were expressed as mean ± SEM. The statistical analysis was performed using one-way analysis of variance (ANOVA) followed by a post-hoc Tukey's test, *p*-values < 0.05 were considered statistically significant.

## SUPPLEMENTARY MATERIALS FIGURES AND TABLES



## Abbreviations

AGE: advanced glycation end products
ALP: alkaline phosphatase
a-SMA: alpha-smooth muscle actin
CaOx: calcium oxalate
DMEM: dulbecco's modified eagle's medium
EMT: epithelial to mesenchymal transition
FBS: fetal bovine serum
HK-2: human renal proximal tubular cells
HPL: trans-4-hydroxy-L-proline
IMCD: inner medullary collecting duct cells
LLC-PK1: porcine proximal tubular cells
MDA: malondialdehyde
MDCK: madin-darby canine kidney
NAC: N-acetyl-L-cysteine
Ox: oxalate ions
PBS: phosphate-buffered saline
RNA: ribonucleic acid
ROS: oxidative stress
RUNX-2: runt-related transcription factor 2
TBARS: thiobarbituric acid reactive substances
TGF-β1: transforming growth factor beta-1
