# Supplementary material for: Oxalate induces type II epithelial to mesenchymal transition (EMT) in inner medullary collecting duct cells (IMCD) in vitro and stimulate the expression of osteogenic and fibrotic markers in kidney medulla in vivo
Source: Oncotarget. 2019 Feb 1;10(10):1102–18. doi: 10.18632/oncotarget.26634 (PMC6383687; doi:10.18632/oncotarget.26634)
Supplement: Supplementary file 1 [file oncotarget-10-1102-s001.pdf]

Oxalate induces type II epithelial to mesenchymal transition (EMT) in inner medullary collecting duct cells (IMCD) *in vitro* and stimulate the expression of osteogenic and fibrotic markers in kidney medulla *in vivo*

SUPPLEMENTARY MATERIALS

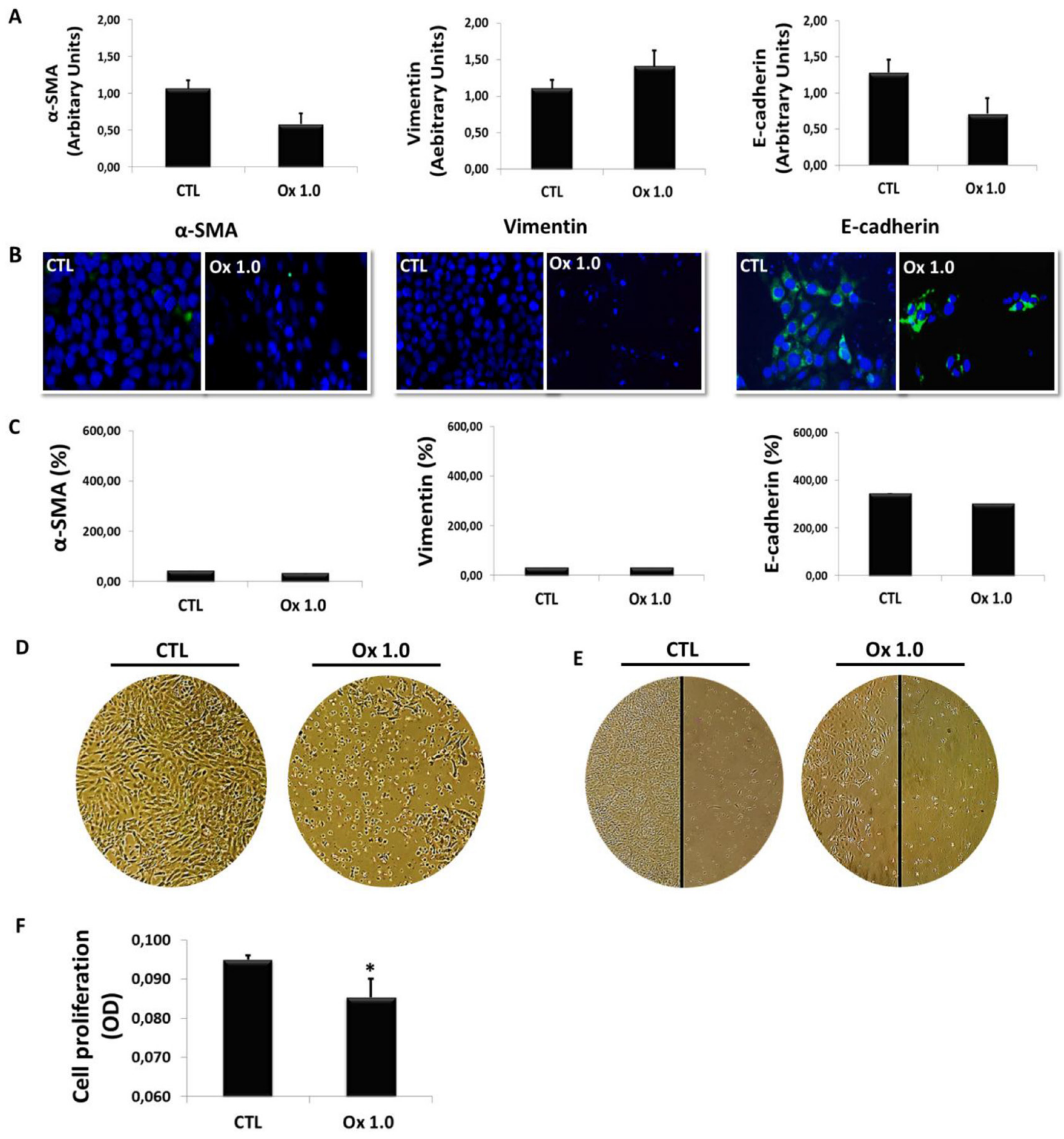

Supplementary Figure 1: IMCD cells in control situation and after 48 h with Oxalate (1.0 mM) exposure. (**A**) The quantitative PCR analysis, (**B**) immunofluorescence images (FITC: green fluorescence, and blue: nuclei) with their respective quantification using ImageJ software (**C**), for α-SMA, Vimentin, and E-cadherin, in both groups were similar. Representative light microscopic images show the presence of few IMCD cells after 48 h with Oxalate (1.0 mM) exposure while CTL group show monolayer growth with clear and rounded delimitations, there was no morphological change (**D**). We did not observe acquisition of migration ability (**E**). Cell proliferation was analyzed by the MTT assay. MTT assay shows that after 48 h with Oxalate (1.0 mM) exposure, we could see a lower cell proliferation of the IMCD cells when compared to the CTL group (**F**). Data are presented as means ± standard errors. (*) significant different when compared to the control group at $p < 0.05$. (ANOVA followed by a post hoc Tukey's test).

Supplementary Table 1: Primers sequence

Gene	Primer	Sequence (5′ to 3′)
E-cadherin 1	5′	ATTCCTGAAGCCCAGGAAATGCAC<br>TGGGCAACTTGGGTTTAAATCG
Vimentin	5′	AGATGGCTCGTCACCTTCGTGAAT<br>TTGAGTGGGTGTCAACCAGAGG
α-SMA	5′	TTGCTGACAGGATGCAGAAGGAGA<br>ATCTGCTGGAAGGTAGACAGCG
β-actin	5′	CCTCTATGCCAACACAGTGC<br>ACATCTGCTGGAAGGTGGAC
TGF-β1	5′	TGGACACCAACTATTGCTTCAGCTCC<br>GAGGTCCTTGCGGAAGTCAATGTA
RUNX-2	5′	AGTTTGCCCTCATCCTTCACTCCA<br>TCAGATACCATGGGTGCTTCTGCT
Alkaline Phosphatase	5′	TTCCTGGCTCTGCCTTTATTCCCT<br>TGGGAATCTGTGCAGTCTGTGTCT
